# Green Synthesis of Hydrogel-Based Adsorbent Material for the Effective Removal of Diclofenac Sodium from Wastewater

**DOI:** 10.3390/gels9060454

**Published:** 2023-06-01

**Authors:** Mariana Chelu, Monica Popa, Jose Calderon Moreno, Anca Ruxandra Leonties, Emma Adriana Ozon, Jeanina Pandele Cusu, Vasile Adrian Surdu, Ludmila Aricov, Adina Magdalena Musuc

**Affiliations:** 1 “Ilie Murgulescu” Institute of Physical Chemistry, 202 Spl. Independentei, 060021 Bucharest, Romania; pmonica@icf.ro (M.P.); aleonties@icf.ro (A.R.L.); jeanina@icf.ro (J.P.C.); laricov@icf.ro (L.A.); 2 Department of Pharmaceutical Technology and Biopharmacy, Faculty of Pharmacy, Carol Davila University of Medicine and Pharmacy, 6 Traian Vuia Street, 020945 Bucharest, Romania; emma.budura@umfcd.ro; 3 Department of Science and Engineering of Oxide Materials and Nanomaterials, Faculty of Applied Chemistry and Materials Science, University Politehnica of Bucharest, 060042 Bucharest, Romania; adrian.surdu@upb.ro

**Keywords:** hydrogel-based materials, adsorbents, removal of pollutants, isotherm, kinetic, diclofenac sodium, water sustainability, adsorption

## Abstract

The removal of pharmaceutical contaminants from wastewater has gained considerable attention in recent years, particularly in the advancements of hydrogel-based adsorbents as a green solution for their ease of use, ease of modification, biodegradability, non-toxicity, environmental friendliness, and cost-effectiveness. This study focuses on the design of an efficient adsorbent hydrogel based on 1% chitosan, 40% polyethylene glycol 4000 (PEG4000), and 4% xanthan gum (referred to as CPX) for the removal of diclofenac sodium (DCF) from water. The interaction between positively charged chitosan and negatively charged xanthan gum and PEG4000 leads to strengthening of the hydrogel structure. The obtained CPX hydrogel, prepared by a green, simple, easy, low-cost, and ecological method, has a higher viscosity due to the three-dimensional polymer network and mechanical stability. The physical, chemical, rheological, and pharmacotechnical parameters of the synthesized hydrogel were determined. Swelling analysis demonstrated that the new synthetized hydrogel is not pH-dependent. The obtained adsorbent hydrogel reached the adsorption capacity (172.41 mg/g) at the highest adsorbent amount (200 mg) after 350 min. In addition, the adsorption kinetics were calculated using a pseudo first-order model and Langmuir and Freundlich isotherm parameters. The results demonstrate that CPX hydrogel can be used as an efficient option to remove DCF as a pharmaceutical contaminant from wastewater.

## 1. Introduction

Fresh water is a unique natural resource, essential for life and particularly precious for the daily existence of humanity and for the surrounding flora and fauna. The global development of the human activities determined by the continuous growth of the population and the increase in pollution are great challenges that require imperative measures regarding the decontamination of water [1]. Pharmaceuticals represent a category of emerging pollutants that have revealed a potential risk to human health and the environment [2,3]. A variety of active ingredients (bisphenol-A, carbamazepine, clofibric acid, diclofenac, ibuprofen, iopamidol, phthalates, polycyclic siloxanes, triclosan) are found in some of the most widely used pharmaceuticals and personal care products (around 3000 registered) [4]. These compounds can contribute to various hormonal abnormalities in humans [5].

Wastewater treatment can be performed in a conventional way, mainly for the removal of the total suspended solids and organic matter [6]. However, following the classical methods, only 94% of the total suspended solids are removed [7]. Therefore, pharmaceuticals can be eliminated by the tertiary steps of adsorption, membrane separation, ozonation, and advanced oxidation processes, or by the use of chemical disinfectants [8,9]. These procedures also have disadvantages because they involve environmentally hazardous chemicals (chemical disinfectants) or high costs. Modern membrane techniques have experienced exponential development in recent years, facilitating the removal of micro- to nano-sized contaminants [10,11] through methods such as electrodialysis, distillation, and direct osmosis [12,13,14,15] using various filtration characteristics (hydrophobicity, surface charge, pore size). Adsorption-based pharmaceutical pollutants removal methods are becoming very attractive for wastewater treatment, presenting advantages such as high efficiency, ease-of-operation mode with fast response on a wide range of adsorbents (natural and artificial), low costs, and non-toxic byproducts. As a result, various adsorption systems based on materials such as clays, alumina, biomass, activated carbon, agricultural waste, silica gel, polysaccharides, and zeolites have been studied and developed [16].

Recent developments in the biosorption processes use many sustainable biomaterials as effective materials in the form of hydrogels, especially natural biopolymers, which are bioavailable, renewable, and biodegradable [17]. Their main advantage is a high adsorption capacity for contaminants from water and friendliness to the environment [18,19]. The 3D polymer networks of hydrogels make them flexible, multifunctional, reusable, and possess good physical and chemical stability [20,21]. They can be adapted to be more efficient in several uses and to increase the adsorption speed, swelling, durability, porosity, and stability [22,23,24].

Among the different biosorbents, chitosan (C) is an abundant and versatile natural biopolymer with an essential contribution for wastewater treatment. It has in its composition two types of monomeric units, one with an amino group and other with an acetamido group, as well as a considerable number of primary amines (–NH_2_) and hydroxyl groups (–OH). These groups provide active sites for the efficient adsorption of anionic and cationic contaminants. The use of chitosan in its native form as an adsorbent has some disadvantages, such as low porosity and strength and a reduced surface area [25,26]. Therefore, the properties of chitosan-based adsorbent materials have been improved through different strategies to overcome these shortcomings [27]. Xanthan gum (X), an extracellular anionic polysaccharide produced by the fermentation of glucose, sucrose, or lactose by the bacterium *Xanthomonas campestris* (a Gram-negative bacterium), is currently used as a thickening agent and emulsion stabilizer due to its thermal stability and pseudoplastic behavior. In addition, it is a biodegradable and biocompatible biopolymer, and it has been widely used to remove contaminants alone and in combination with other natural or synthetic polymers [28].

Although in the literature there are many materials used for the adsorption of pharmaceuticals from wastewater [29,30,31,32], the scientific novelty of the present research is the design of a green hydrogel as an economic adsorbent, easy to be prepared and easy to be modified, and cost-effective, but with high efficiency for removing the pharmaceutical contaminants from water. For this purpose, diclofenac sodium was chosen as a “model-drug system”. In this regard, to achieve a hydrogel-based adsorbent with suitable properties and a good performance, chitosan, polyethylene glycol 4000 (PEG4000), and xanthan gum were chosen to achieve a crosslinked hydrogel network.

Worldwide, the global consumption of diclofenac (DCF), especially used in the treatment of inflammation and pain [33], has been estimated at approximately 940 tons per year, from which approximately 65% of the oral dose of this drug is released through urine and feces, along with its active metabolites, and passes through conventional wastewater treatment plants [34]. DCF is also found in waters due to improper disposal as solid waste or due to ineffective conventional treatments through effluents from industrial and urban wastewater treatment plants [35]. The European Commission defined limits of chronic toxicity with respect to the annual average and acute toxicity for DCF, establishing the maximum allowable concentrations between 0.1 and 0.01 μg/L for surface inland waters and between 75 and 7.5 μg/L for coastal waters [8,36]. Recently, eliminating DCF from the aqueous environment has become a challenge for the scientific community, especially in the context of the establishment by the United Nations organization of the positive impact on many of the Sustainable Development Goals, due to its long-term importance for people and the environment [6].

Adsorbent materials based on various hydrogels have been developed and studied to remove sodium diclofenac from water. A hydrogel composed from bio-based egg albumin (ALB) functionalized with a high density of amine adsorption sites via polyethyleneimine (PEI) demonstrated excellent DCF removal capacity, i.e., 232.5 mg/g under optimal experimental conditions (pH~6; contact time~180 min; adsorbent dose~0.5 g/L) [37]. A poly(methacrylic acid)/montmorillonite (PMA/nMMT)-based nanocomposite hydrogel showed good adsorption capacity for the removal of amoxicillin (152.65 mg/g) and diclofenac (DCF) (152.86 mg/g) and an efficient regeneration [38]. Macroporous chitosan hydrogels were synthesized by crosslinking with genipin and incorporated n-GO as effective adsorbents for DCF. The addition of n-GO has promoted the DCF adsorption process and led to 100% removal of DCF after only 5 h [39]. Self-assembled reduced graphene oxide (rGO) three-dimensional hydrogels demonstrated a removal efficiency of naproxen (NPX), Ibuprofen (IBP), and diclofenac (DCF) between 70 and 80% at acidic pH and showed fast adsorption kinetics [40]. GO nanoparticles were shown to act as both adsorbents and crosslinking agents [41]. Reduced graphene oxide magnetite (r-GOM) has also demonstrated efficacy in removing diclofenac sodium (5.249 mg/g adsorption capacity) and aspirin (23.59 mg/g) from wastewater [42].

Here, the green synthesis of a hydrogel based on crosslinked chitosan and PEG4000 and using xanthan gum as a thickening agent was pursued as a new environmentally friendly, efficient adsorbent material used for the removal of DCF from aqueous media. Furthermore, the physical, chemical, rheological, and pharmacotechnical parameters were determined, and the adsorption kinetics of DCF were analyzed to evaluate the drug adsorption efficiency from the aqueous media.

## 2. Results and Discussion

### 2.1. Visual Examination

**Figure 1:**
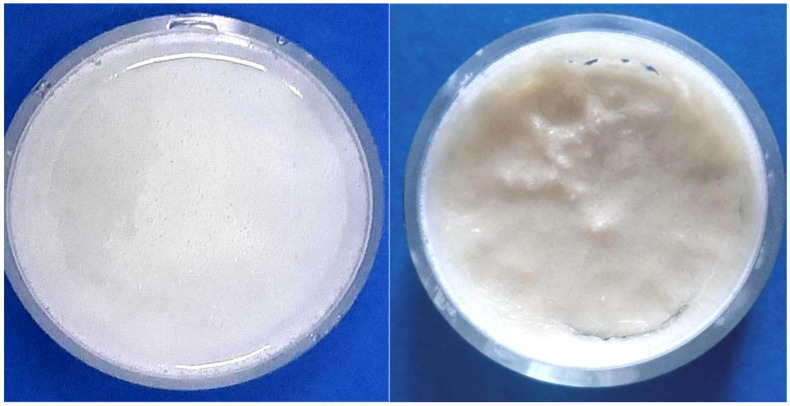
Optical images of the CPX hydrogel: wet (**left**) and dry (**right**).

The hydrogel formation capability of xanthan gum is well known. Complex gel polymers of chitosan and xanthan gum with enhanced properties have been reported previously [43,44,45,46,47,48,49]. Figure 1 shows the CPX hydrogel obtained after the reaction between chitosan, xanthan gum, and PEG4000, as prepared (wet) and dry. The dry hydrogel is yellowish–white and has a gelatinous aspect. The complexation reaction between chitosan, xanthan gum, and polyethylene glycol occurs due to interactions among the opposite charges presented in the biopolymers: NH_3_^+^ groups of chitosan, COO^−^ groups of xanthan gum, and HO^−^ end-groups from PEG chains. When chitosan, xanthan gum, and polyethylene glycol are mixed together in solution, they can form a complex through electrostatic interactions among the positively charged chitosan and the negatively charged xanthan gum and PEG. The complexation process can have as a result the changes in the properties of each component from the mixture (hydrogel matrix). The interaction among chitosan, xanthan gum, and PEG can help to strengthen the hydrogel structure, leads to an increase in viscosity due to the formation of a three-dimensional polymer network, and improves its chemical and mechanical stability, as well as its adhesive properties. 

### 2.2. Infrared Spectroscopy Measurements

**Figure 2:**
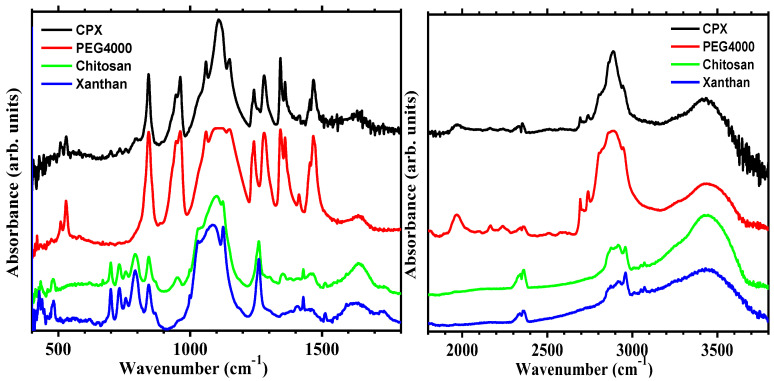
FTIR spectra of chitosan, xanthan gum, PEG4000, and the developed CPX hydrogel.

FTIR spectra of chitosan, xanthan gum, PEG4000, and the developed hydrogel CPX are shown in Figure 2.

The main vibrational bands observed in the FTIR spectra of each individual component include O–H stretching vibrations observed by the broad band at around 3500–3200 cm^−1^, corresponding to the stretching of the hydroxyl groups (–OH) present in the polymer chain for all compounds (red, green, and blue lines). Similar bands were noticed in the spectra of the polyethylene glycol 4000, xanthan gum, and chitosan previously reported [50,51,52,53,54,55,56,57]. C–H stretching vibrations were noticed by the band at around 3000–2800 cm^−1^, corresponding to the asymmetric and symmetric stretching of the carbon–hydrogen (C–H) bonds present in the (–CH_3_) groups, in agreement with previous reported bands observed in chitosan, xanthan gum, and PEG4000 [50,51,52,53]. C=O stretching vibrations registered by the band at around 1650 cm^−1^ in the chitosan spectrum (green line) corresponds to the stretching of the carbonyl group (C=O) and is relatively weak compared to the other peaks in the spectra, as also observed by Zajac et al. [50] and de Morais et al. [53] in chitosan. C–C and C–O–C bending vibrations are represented by the bands at 1450–1470 cm^−1^, which correspond to the bending of the carbon–carbon (C–C) and carbon–oxygen–carbon (C–O–C) linkages. Weak bands in the same region were also noticed by Zajac et al. [50] and Nirmala [55]. C–H bending vibrations are assigned to the weak bands at around 1410 cm^−1^ and 1340–1360 cm^−1^, and they correspond to the bending of the carbon–hydrogen (C–H) bonds. Bands corresponding to C–H bending vibrations were observed at 1422 cm^−1^ and 1325–1377 cm^−1^ by Zajac et al. [50]. The band observed at 1280 cm^−1^ is typically assigned to the bending vibrations of the carbon–hydrogen (C–H) bonds present in the methylene (–CH_2_–) groups in the PEG backbone (red line). This band was observed at 1262 cm^−1^ by Zajac et al. [50]. C–O–C asymmetric stretching vibrations: the peak at around 1240 cm^−1^ is characteristic of the asymmetric stretching of the ether linkages (–O–) present in the PEG molecule. The C–O–C and C–O stretching vibrations are represented by the intense peak at around 1100 cm^−1^, corresponding to the stretching vibrations of the carbon–oxygen (C–O) bond present in the ether linkages (–O–). The 1150 cm^−1^ peak corresponds to the asymmetric stretching of the C–O bond and is often referred to as the “C–O stretching” peak. This band position correlates with other published spectra of the same polymers [56,57]. The intense peaks indicate the presence of multiple ether linkages in the polymer chain. The peak at around 1040 cm^−1^ corresponds to the stretching of the carbonyl groups (C=O) present and is relatively weak compared to the C–O–C stretching band. C–O–C rocking vibrations: the intense peak at 850 cm^−1^ corresponds to the rocking of the ether linkages (–O–) present in the PEG molecule (red line) and chitosan (green line), observed at 896 cm^−1^ by Zajac et al. [50] and Dey et al. [52]. In summary, the IR spectrum of PEG4000 (red line) is characterized by intense and broad O–H stretching vibrations, intense C–O–C stretching vibrations, and C–H and C=O stretching and bending vibrations.

The synthesized hydrogel spectrum (black line) exhibited some bands with slight shifting and lower intensities compared with the PEG4000 spectrum, especially in the main bands region in 2800–3000 cm^−1^, 1000–1200 cm^−1^, and 600–800 cm^−1^, overlapping (in the 500–600 cm^−1^, 800–1000 cm^−1^ regions), or the appearance of some characteristic peaks of pure components in the developed polymeric network (in 800–1000 cm^−1^ and 1200–1350 cm^−1^, 2700–2800 cm^−1^), which is a clear indication of interaction among the components through intramolecular re-arrangement, hydrogen bonding, or alteration in the positions of functional groups in the final hydrogel structure. 

### 2.3. Raman Spectroscopy Results

**Figure 3:**
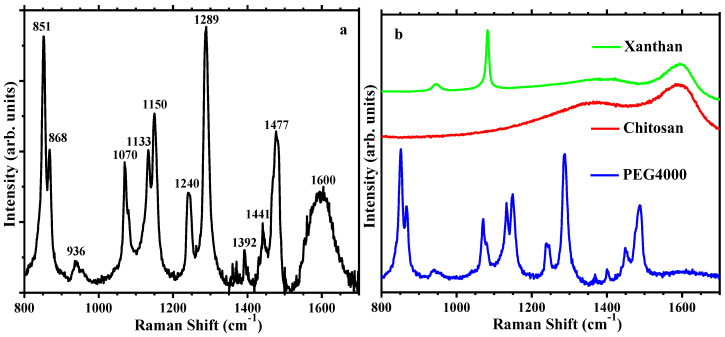
The Raman spectra of the CPX hydrogel (**a**) and the Raman vibration modes of the gel components (**b**).

As shown in Figure 3, the Raman spectra of the CPX hydrogel (Figure 3a) present well-defined bands in the 800–1700 cm^−1^ spectral region that correspond to the vibration modes of the gel components, shown in Figure 3b. The majority of the bands in Figure 3a correspond to PEG4000, with a stronger Raman signal. The presence of chitosan or xanthan gum is revealed by the wide Raman band centered at 1600 cm^−1^; the G band from C–C bonds in the backbone of the polymeric structure, which is not present in the Raman spectra of PEG4000; and a shoulder at 1082 cm^−1^ from xanthan gum. The peak at 851 cm^−1^ is related to the superposition of a number of vibrations with the main contributions of the CH_2_ rocking, C–O stretching, and C–C stretching vibrations. Therefore, the peak positions of the Raman bands at about 851, 1140, and 1477 cm^−1^ can serve as a quantitative measure of the molecular weight distribution for short PEG molecules. Their positions confirm molecular weights over 2000 [58]. According to Matsuura [59,60], the bands at 868, 936, 1070, 1133, and 1150 cm^−1^ are also assigned to the superposition of a number of vibrations with the main contributions of the CH_2_ rocking, C–O stretching, and C–C stretching of terminal groups, while the bands observed at higher Raman shifts are assigned to CH_2_ modes: in-plane twisting at 1289 cm^−1^, out-of-plane wagging at 1392 cm^−1^, and scissoring vibrations at 1441 and 1477 cm^−1^ [59,60,61].

### 2.4. XRD Results

**Figure 4:**
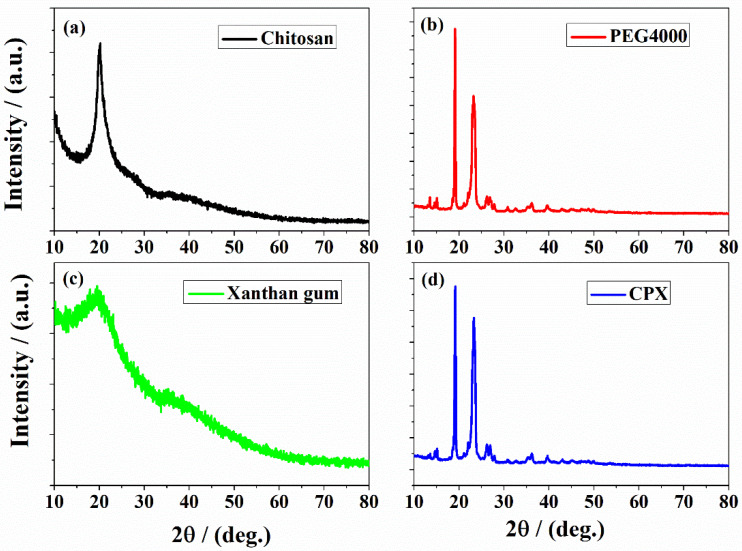
XRD diffractograms of (**a**) chitosan, (**b**) PEG4000, (**c**) xanthan gum, and (**d**) the developed CPX hydrogel.

The X-ray diffraction method was used to examine the structure of CPX hydrogel. The XRD patterns of the raw materials (chitosan, PEG4000, and xanthan gum) and the obtained CPX hydrogel are shown in Figure 4.

The X-ray pattern of chitosan (Figure 4a) shows an intense reflection from (200) planes at 2θ = 20.2°, revealing a crystalline structure [62,63]. The pattern of PEG4000 (Figure 4b) displays two sharp and strong diffraction peaks at approximately 19.6° and 23.4° [64,65]. Xanthan gum (Figure 4c) presents no sharp peaks, indicating its amorphous structure, in agreement with the earlier reports [21,66]. The X-ray pattern of the CPX hydrogel (Figure 4d) is dominated by the intense diffraction peaks of PEG4000; however, the peaks are slightly shifted and with modified intensities, as a result of the interaction among the three components in the hydrogel network. Therefore, the observed XRD pattern is in good agreement with the findings observed in the FTIR analysis.

### 2.5. Differential Scanning Calorimetry (DSC) Analysis

**Figure 5:**
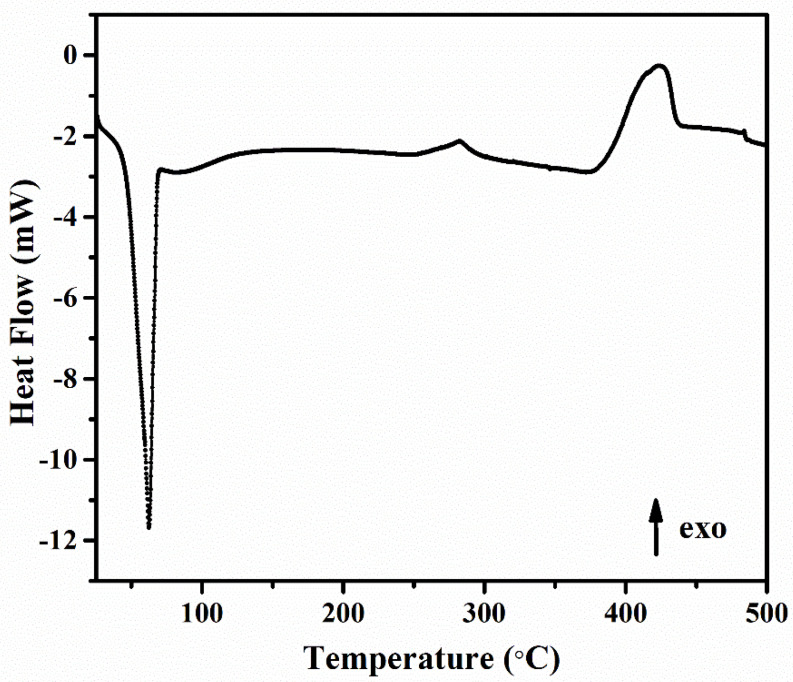
The DSC thermogram of the CPX sample.

Figure 5 shows the DSC thermogram of the CPX hydrogel. 

The first thermal event from Figure 5 is a wide endothermic peak centered between 51 and 68 °C, with an onset at 51 °C. The values for the temperature and associated enthalpy of the peak minimum are 62 °C and 577.08 mJ. The endothermic peak is associated to dehydration, the loss of water associated with the hydrophilic groups of chitosan [67,68]. In the solid state, chitosan-based polysaccharides have disordered structures and have a strong affinity for water and, as a result, can be easily hydrated [69]. The hydration properties of these polysaccharides depend on the primary and supramolecular structures [70,71]. This peak indicates that the sample was not completely dried, and there was some bound water, which was not removed during drying. Appelqvist et al. [72] and Gidley and Robinson [73] reported an endothermic dehydration peak in a similar range of 60 ± 10 °C for a range of polysaccharides at a moisture level between 5 and 25%, which they attributed to the enthalpic association between water and carbohydrate.

The DSC thermogram of the sample also showed a small exothermic event between 270 and 300 °C, which can be attributed to scissions in the polymeric network of polysaccharides [68]. The wide exothermic peak between 370 and 430 °C can be attributed to the overall decomposition of highly substituted regions in polysaccharides. The exothermic peak is assigned to the thermal degradation of the composite polymeric material (monomer dehydration, glycoside bond cleavage, and decomposition of the acetyl and deacetylated units) [74,75].

Thus, the observed DSC events are in good agreement with the thermal decomposition profiles observed by TGA.

### 2.6. Thermogravimetric and Differential Thermal Gravimetric Analysis

**Figure 6:**
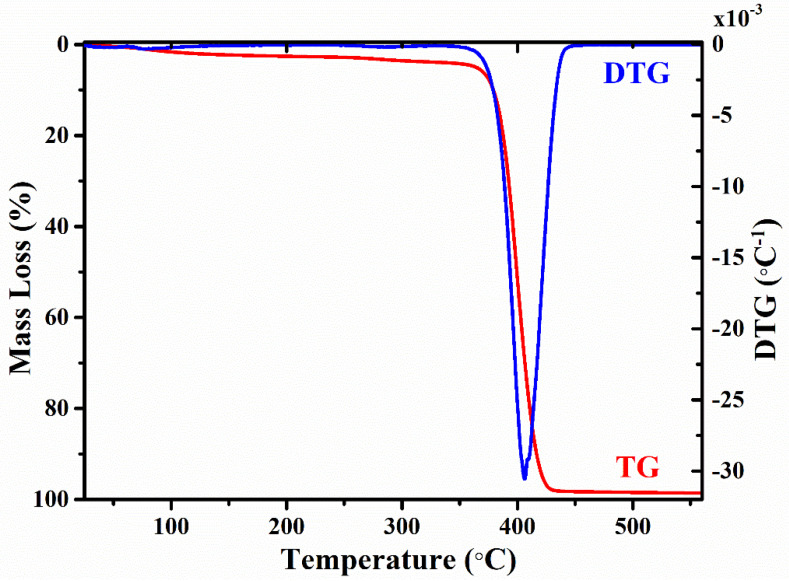
Thermogravimetric and differential thermal gravimetric analysis of the CPX hydrogel.

Figure 6 shows the TG/DTG curves of the CPX hydrogel. 

A small (<5%) weight loss is observed in the TG curve between 20 and 100 °C, associated to the evaporation of water (moisture loss), being a continuous, progressive weight loss, confirmed by the DTG curve. After dehydration, no further weight loss is observed up to 370 °C, indicating a good thermal stability of the polymeric network. A substantial weight loss occurs between 370 °C and 430 °C, indicating the complete pyrolysis of the polymeric composite (chitosan, xanthan gum, and PEG4000) in a single thermal event. TG measurements reveal a total mass loss of 99.37% at 550 °C, which confirms the full decomposition of the composite polymer. Similar results were also reported [53,76,77].

A plateau of thermal stability was observed after the thermal decomposition of the organic material of the components, whose weight loss started before finishing the dehydroxylation step. These results are compatible with the process of thermal degradation of the polymeric network [78,79]. During the pyrolysis, a random split of the glycosidic bonds occurs in the chitosan and PEG network, which is further followed by decomposition forming acetic, butyric, and fatty acids [52].

### 2.7. SEM Analysis

**Figure 7:**
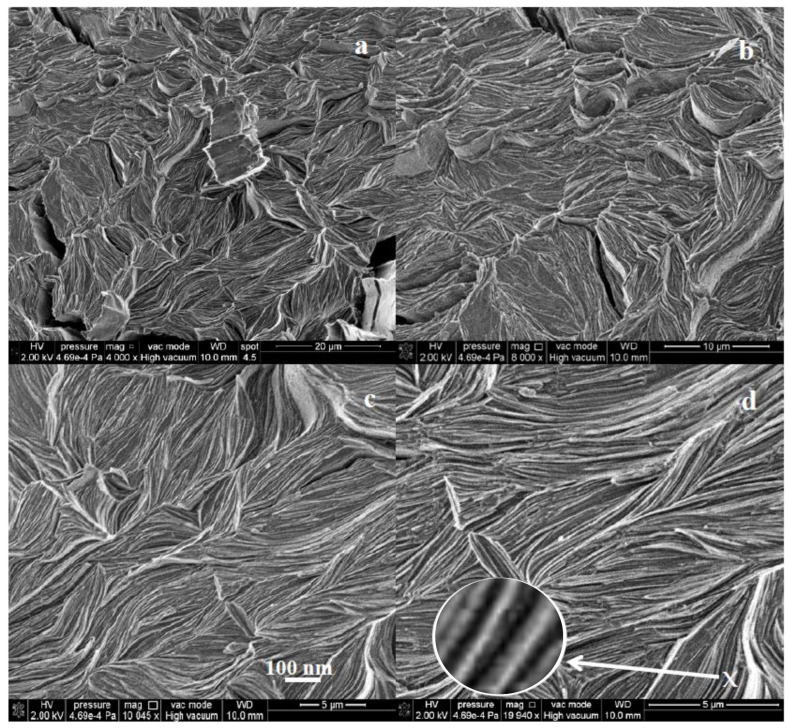
SEM micrographs at different magnifications showing the microstructure of the CPX hydrogel. (**a**,**b**) bundles of two-dimensional stacked sheet-like structures with lengths of tens of microns, (**c**,**d**) magnified images with thicknesses in the nanorange (below 100 nm); a magnified image (300,000×) at the edge of a bundle of stacked nanosheets, at the position marked with an ‘x’.

SEM images of the dried CPX hydrogel show a fibrillar morphology (Figure 7), consisting of bundles of two-dimensional stacked sheet-like structures (Figure 7a,b), with lengths of tens of microns, as shown in more detail in Figure 7c–d, and thicknesses in the nanorange (below 100 nm), illustrated in the inset of Figure 7d, a magnified image (300,000×) at the edge of a bundle of stacked nanosheets, at the position marked with an ‘x’. The combination of negatively charged (PEG with OH^-^ groups and xanthan gum with COO^−^) and positively charged (chitosan with NH^3+^ groups) electrolytes is known to induce intimate mixing in ordered morphologies. Chitosan–xanthan gum composite hydrogels have previously been shown to produce two-dimensional structures [80,81]. These two-dimensional ordered structures are highly advantageous for enhancing the swelling capacity of hydrogels by filling and expanding the cavities between individual sheets, while maintaining the structural integrity of the polymer network.

### 2.8. Rheology

**Figure 8:**
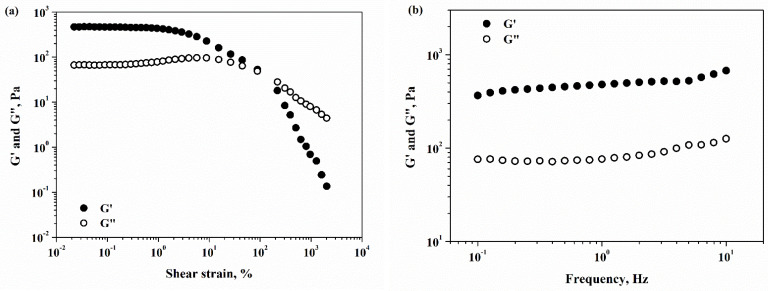

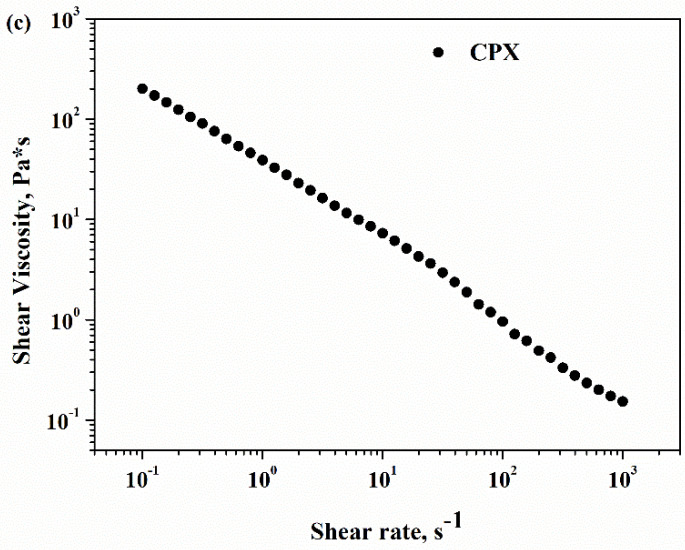
Rheological characterization of CPX hydrogel: (**a**) Storage and loss moduli vs. shear strain; (**b**) Storage and loss moduli vs. frequency; and (**c**) Shear viscosity vs. shear rate.

The gel nature of the obtained hydrogel was confirmed using rheology analysis. The dynamic rheological behavior of the CPX hydrogel was investigated, and the results are presented in Figure 8.

The CPX hydrogel (Figure 8a) showed a linear viscoelastic response at a shear strain less than 2%, with G′ and G″ being independent of applied strain, and the elastic modulus being 7 times greater than the viscous one. After a 2% strain stress, the non-linear viscoelastic behavior occurs, although G′ remains the dominant part. After that, the elastic modulus considerably decreases, and the viscous component takes control of the nonlinear behavior at a strain amplitude of about 80% (the crossover point). The dependences of rheological moduli on applied frequency at 0.5% stress strain are illustrated in Figure 8b. The CPX hydrogel responded to frequency measurements with a gel-like response, with G′ higher than G″, and both rheological moduli appear to be almost frequency-independent. The same behavior was observed for other hydrogels used for adsorption of pharmaceuticals from water [31]. Further, for the CPX hydrogel, the shear viscosity decreases as the shear rate increases (Figure 8c), pointing to a non-Newtonian pseudoplastic fluid with shear-thinning properties.

### 2.9. Pharmacotehnical Characterization

The dry CPX hydrogel showed a tensile strength of 0.84 ± 0.09 kg/mm^2^ and 17.04 ± 0.12% elongation. The low moisture content, 5.22 ± 0.43%, explains the reduced hardness and flexibility, as expected for dehydrated hydrogels that contain little or no water.

**Figure 9:**
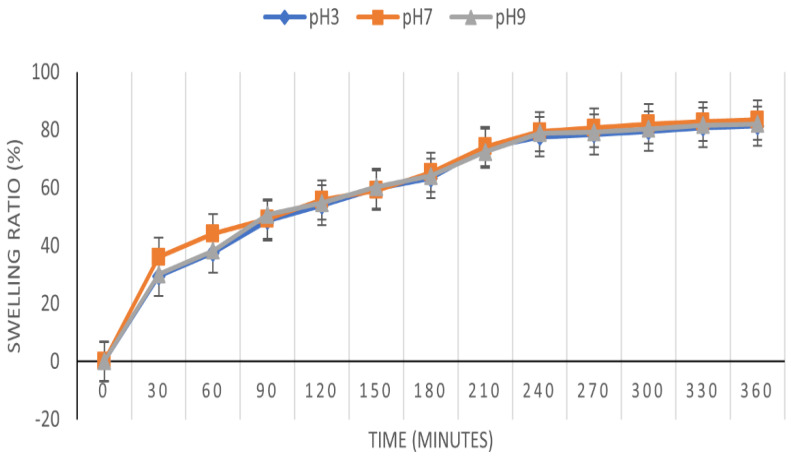
The CPX hydrogel swelling degree over 6 h at pH 3, pH 7, and pH 9.

The CPX hydrogel swelling degree over 6 h in 3 different media is shown in Figure 9. Swelling performance is affected by different parameters, such as the hydrophilicity and hydrophobicity of the polymers type, crosslinking density of the hydrogel network, and pH conditions [82].

The CPX hydrogel swelling behavior is almost similar at different pH values, proving that it is not pH-sensitive, and has the same performance in any medium. Similar results were reported in the literature [43,83,84]. These studies described that the hydrogel network, which was designed through the ionic interactions among the amino groups from chitosan and the carboxyl groups from xanthan gum in the hydrogel matrix, which facilitates the controlled adsorption of various molecules.

The swelling degree was demonstrated to be developed linearly for the first 4 hours (83%), after which the increase slowed down, with the differences between 240 and 360 min being essentially unimportant. The findings show that independent of pH conditions, the swelling ability is the highest in the first 30 min.

### 2.10. Preliminary Adsorption Studies 

**Figure 10:**
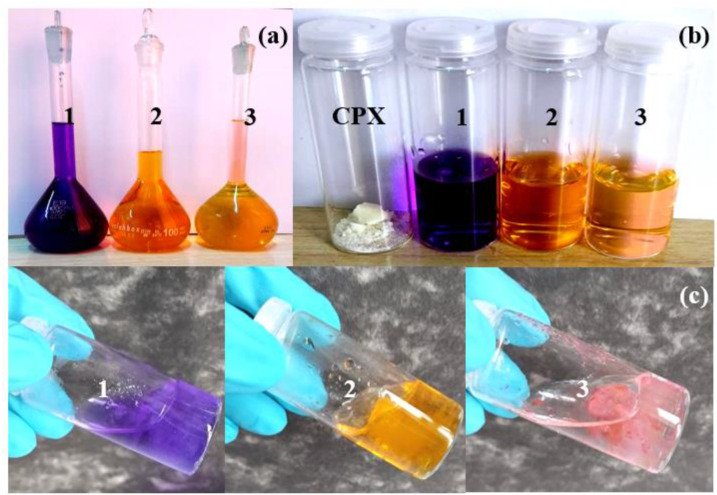
Visual dye preliminary adsorption tests of CPX hydrogel. (1) Gentian violet; (2) methyl orange; and (3) eosin; (**a**) stock solution; (**b**) initial time; (**c**) after 24 h.

Preliminary investigation of the adsorption tests was made using various dyes (Figure 10a; gentian violet, methyl orange, and eosin), and the results are depicted in Figure 10. A color change from the initial colors (Figure 10b) to lighter shades (Figure 10c) after 24 h of contact time was visually observed, indicating that the dye was easily adsorbed by the hydrogel.

### 2.11. Batch Adsorption Study

**Figure 11:**
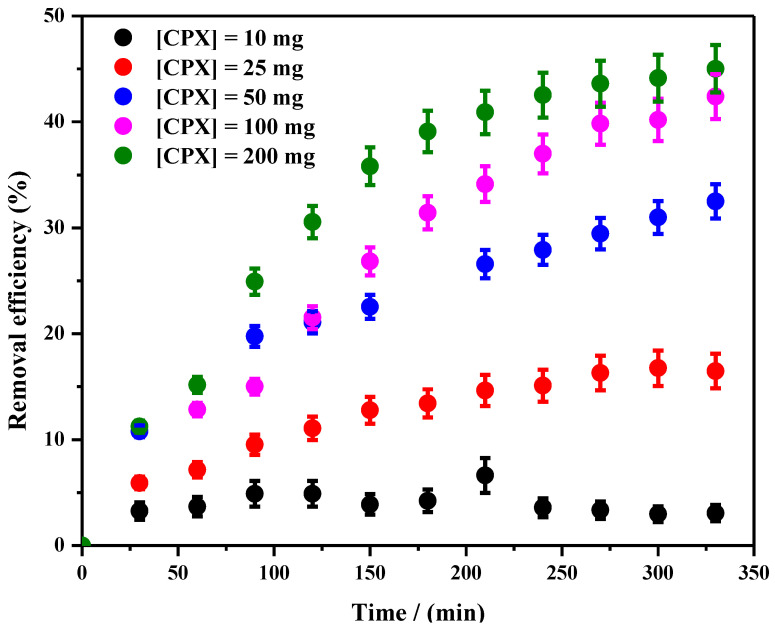
Effect of contact time on DCF adsorption on CPX hydrogel over time.

The effect of contact time on the adsorption of DCF on the CPX hydrogel is presented in Figure 11. 

**Figure 12:**
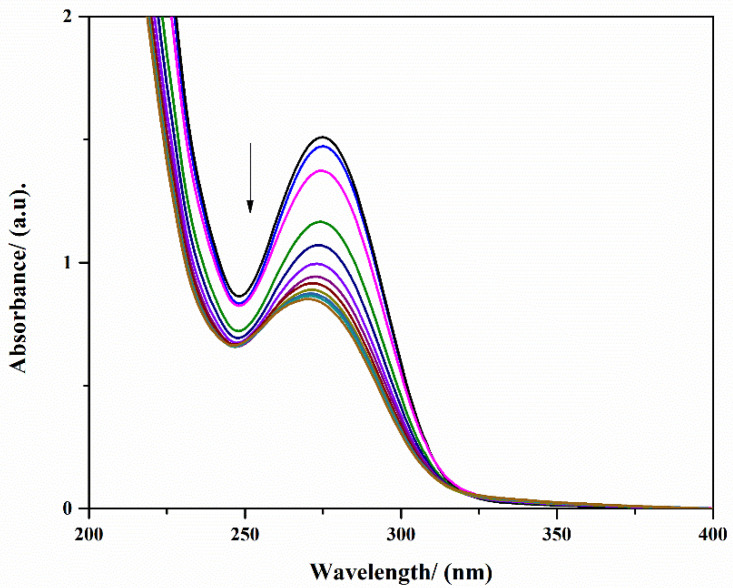
UV–Vis spectra of adsorption of DCF in presence of 50 mg CPX hydrogel during 330 min.

It was observed that the DCF is slowly adsorbed into the CPX hydrogel after 350 min, reaching 45% in the highest adsorbent amount (200 mg) (Figure 11). During the adsorption study, no blue or red shifting of the characteristic adsorption maximum of DCF was observed. Since for all tested CPX hydrogel amounts, the behavior was quite similar, Figure 12 shows an exemplification of the adsorbance variation in the considered time frame.

When chitosan, xanthan gum, and polyethylene glycol are mixed together in solution, they can form a complex through electrostatic interactions among the positively charged chitosan and the negatively charged xanthan gum and PEG. PEG4000 is considered to be hydrophilic and is soluble in aqueous solutions. This is because PEG is a polar molecule, with hydroxyl groups (–OH) along its polymer chain that can interact with water molecules through hydrogen bonding. The hydrophilic properties of PEG can vary, depending on the length of the polymer chain; shorter chains of PEG are more hydrophilic, while longer chains can become more hydrophobic due to the increased number of non-polar carbon atoms in the polymer backbone. Nevertheless, PEG4000 is still generally considered to be a hydrophilic compound. Xanthan gum contains numerous hydrophilic functional groups, such as hydroxyl (–OH) and carboxyl (–COOH) groups. As a result, xanthan gum is highly soluble in water and widely used as a thickener, stabilizer, and emulsifier. Chitosan contains hydroxyl and amino groups that are hydrophilic and can interact with water molecules through hydrogen bonding, as well as acetyl and amino groups that are hydrophobic and can interact with non-polar molecules through van der Waals interactions. DCF is a polar compound that contains both hydrophilic and hydrophobic functional groups. It has a carboxylic acid (–COOH) group and a phenolic (–OH) group that are both hydrophilic and can interact with water molecules through hydrogen bonding. However, it also has two chloro (–Cl) substituents that are hydrophobic and can interact with non-polar solvents through van der Waals interactions. Therefore, both electrostatic and hydrophobic interactions may contribute to the adsorption of DCF.

**Figure 13:**
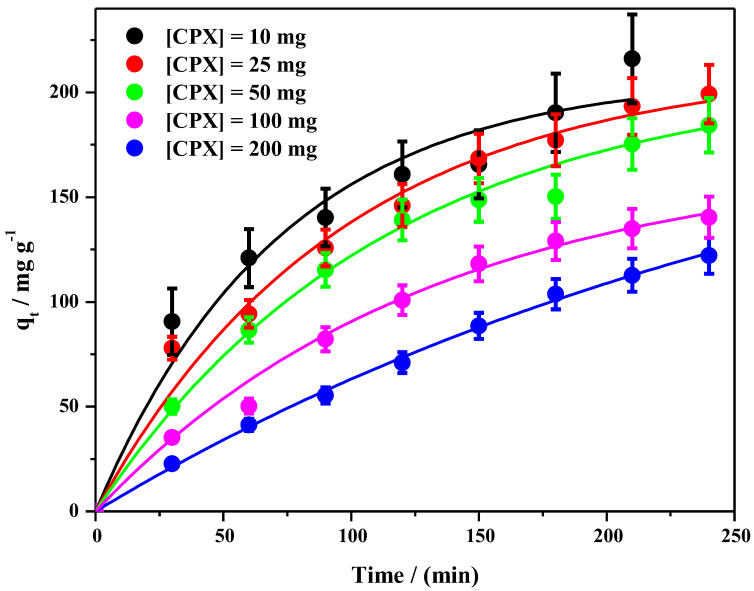
The plot of the non-linear form of the pseudo first-order model for different amounts of CPX hydrogel and 0.33 mg DCF [Solid lines represent the best fit of the experimental data points using Equation (10)].

Subsequently, the pseudo first-order model was employed to fit the experimental data in order to study the DCF adsorption process (Figure 13).

Meanwhile, Table 1 contains the corresponding model parameters of the fits for a pseudo first-order kinetics. The pseudo first-order model assumes that the adsorption rate is limited mainly by the diffusion step [85,86].

The pseudo second-order model for the adsorption of DCF in different amounts of CPX hydrogel was also analyzed. The model was fitted using a linear equation. The results, together with the errors, are shown in Figure S1 and Table S1 from the Supplementary Materials. The adjusted R square is much lower than 1, suggesting that the adequate model for the investigated systems containing DCF follows a pseudo first-order model. 

Since the adsorption takes place at equilibrium, the experimental data were analyzed by Langmuir [86] and Freundlich [87] models, and the type of adsorption that takes place between the aqueous DCF species and adsorption sites was determined (Figure 13). Table 2 provides the linear correlation coefficients (R^2^) and the isotherm constants (q_max_, b, n, and K_F_) for the Langmuir and Freundlich models. In addition, the equilibrium parameter (R_L_) was calculated. The Langmuir and Freundlich isotherm graphical representation is shown in Figures S2 and S3 from the Supplementary Materials. 

Obviously, the R^2^ value of the Langmuir model (0.99) is substantially higher than that of the Freundlich model (0.93) and close to 1.0. Furthermore, the theoretical value of q_max_ (172.41 mg/g) is in agreement with the experimental data. The R_L_ parameter is lower than 1, which suggests that DCF adsorption is a favorable phenomenon. Similar results were reported in the literature using different hydrogel-based adsorbent materials for DCF adsorption studies [88,89]. The Langmuir model showed that the DCF adsorption process on the CPX hydrogel matrix was close to monolayer adsorption [81]; the adsorption sites were uniform and independent [81], with no interaction involving adsorption molecules [90].

The kinetic analysis indicates that the DCF adsorption process tends to follow a Langmuir-type adsorption. Therefore, it is likely that the adsorption of DCF and dyes involves mainly electrostatic interactions, governed by hydrogen bonds, with some contribution of Van der Waals forces.

The data obtained in the kinetic study reveal that the used adsorbent may have a high potential to be used in environmental applications as a water cleaning agent.

## 3. Conclusions

A green adsorbent hydrogel based on chitosan, polyethylene glycol 4000, and xanthan gum was synthesized using a simple and easy aqueous solution method. The FTIR, Raman, XRD, DSC, and TGA analyses confirmed the occurrence of interactions among the three components, through the intramolecular rearrangement of hydrogen bonds and in the modification of the positions of the functional groups, in the final hydrogel assembly. The SEM analysis evidenced a two-dimensional ordered structure that has a great advantage in improving the swelling capacity of CPX by approximately 83% after 360 min and does not depend on the pH medium.

Preliminary adsorption tests showed that CPX has the capacity to adsorb different dyes (gentian violet, methyl orange, and eosin) from water after 24 h of contact time, evaluated by visual observation. The adsorption ability of the CPX hydrogel towards the removal of pharmaceuticals from water was tested for DCF as the tested drug. CPX hydrogel exhibited a remarkable adsorption capacity (172.41 mg/g) for DCF. For the tested drug, the adsorption kinetics were found to follow the pseudo first-order model, while the adsorption mechanism was explained by the Langmuir and Freundlich models. Taken together as a first report, these results allow us to conclude that CPX-based adsorbent hydrogel can be used as a promising facile, ecological, and cost-effective adsorbent for the removal of pharmaceuticals from wastewater.

## 4. Materials and Methods

### 4.1. Chemicals and Reagents

All materials were of analytical grade. Chitosan (C) (deacetylated chitin, poly(D-glucosamine), medium molecular weight M.W. = 190,000–310,000 (MMWCH) and viscosity = 200–800 cps in 1% acetic acid, degree of deacetylation 75–85%), acetic acid (≥99.7%), and polyethylene glycol of molecular weight 4000 (PEG4000) were purchased from Merck, Germany. Elemental SRL, Romania, supplied xanthan gum (X). The experiments were performed with deionized (DI) water (resistivity of 18.2 Ω⋅cm at 25 °C). All reagents were used as received, without further purification.

### 4.2. CPX Hydrogel Preparation

**Figure 14:**
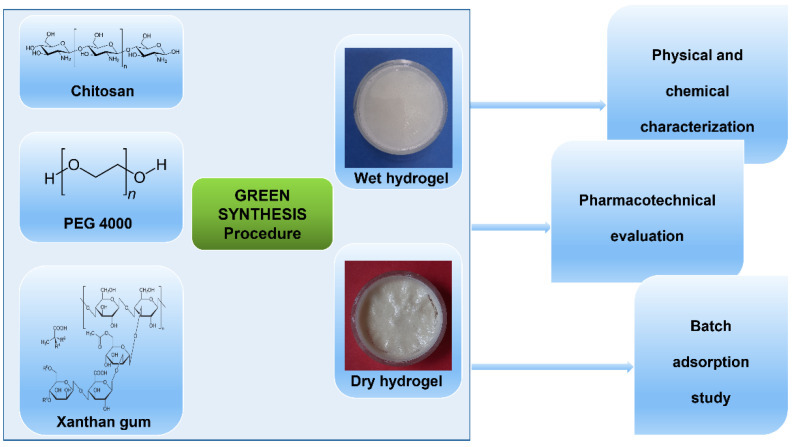
Schematic illustration of the synthesis procedure and the evaluation of the hydrogel-based adsorbent material.

The hydrogel was prepared by a simple and easy mechanical mixing method in aqueous solution and with a final light heat treatment, by using the minimum quantities of materials able to promote the formation of hydrogel matrix. Firstly, 100 mL of an aqueous solution containing 1% chitosan (*w*/*v*) in 1% acetic acid (*w*/*v*) was prepared and stirred overnight until the chitosan was completely solubilized. Subsequently, 40% PEG4000 was added to this solution under vigorous continuous stirring, at 800 rpm, for approximately 1 h for homogeneous mixing. Next, 4% xanthan gum was added to the solution, under stirring, and slightly warmed below 40 °C for approximately 30 min, until gelation occurred. Finally, the obtained hydrogel, (notation: CPX, for its chitosan (1%)—PEG4000 (40%)—xanthan gum (4%) composition) was left to rest at room temperature until the next day. After being organoleptically examined, the synthesized CPX hydrogel was poured into Petri dishes and kept until completely dry. Figure 14 presents a schematic illustrating the synthesis and characterization of the hydrogel-based adsorbent material.

### 4.3. Methods

#### 4.3.1. Visual Examination

The obtained hydrogel was examined regarding its specific properties, such as color, appearance, homogeneity, consistency, and phase separation, or the presence of agglomerations [21]. 

#### 4.3.2. Physical and Chemical Analysis

Fourier Transform Infrared (FTIR) analysis was carried out using a Nicolet 6700 apparatus in the range of 4000–400 cm^−1^. Potassium bromide KBr of spectroscopic grade was used for mixing the samples. The measurements were obtained in absorbance mode, with a sensitivity of 4 cm^−1^. For further qualitative analysis, the normalized spectra were used.

A Horiba Jobin Yvon LabRam HR spectrometer (Horiba, Ltd., Kyoto, Japan) was used for recording the Raman spectra. An excitation laser at 325 nm and a NUV 40× objective, using an integration time of 60 s, was utilized.

XRD spectra were obtained using a PANalytical Empyrean diffractometer with a Cu X-ray tube (λ Cu Kα1 = 1.541874 Ǻ), at room temperature. X-ray diffractograms were collected using a 0.02° scan step, in the range of 20°–80°, in a Bragg–Brentano geometry.

Differential scanning calorimetry (DSC) analysis was recorded with a Mettler Toledo DSC 3 calorimeter and carried out to obtain the DSC curves, in a nitrogen atmosphere with a gas flow of 80 mL min^−1^. The samples were sealed in crimped Al pans with a pinhole in the lid. 

Thermogravimetric (TG/DTG) analysis was achieved with a Mettler Toledo TGA/SDTA851^e^ instrument, in a synthetic airflow atmosphere with a flow of 80 mL min^−1^, using 70 µL open alumina pans. The used heating rates for DSC and TG/DTG analyses were 10 °C/min.

Scanning electron microscopy (SEM) was carried out to study the morphology of dry hydrogel in a Quanta 3D field emission microscope in secondary electron images, which operate in high vacuum mode, at an accelerating voltage of 2 kV.

The rheological measurements were performed on a Kinexus Pro rheometer at 25 °C, with a 0.8 mm gap using parallel plate geometry. The linear viscoelastic domain of storage and loss moduli (G′ and G″) was determined at a constant frequency (1 Hz), as a function of shear strain. The G′ and G″ were evaluated as a function of frequency (0.1–10 Hz) in the linear viscoelastic regime at a shear strain of 0.5%. Moreover, the shear viscosity was investigated at applied shear rates ranging from 0.1 to 1000 s^−1^. Rheological data were presented using a logarithmic scale.

#### 4.3.3. Pharmacotechnical Characterization and Elongation Ability and Tensile Strength

The mechanical performance was tested with a digital tensile force tester used for universal materials, produced by Lloyd Instruments Ltd., LR 10K Plus (West Sussex, UK). Between the two plates positioned at a distance of 30 mm, the dry hydrogel was placed vertically, and the breaking force was measured at a speed of 30 mm/min. The mechanical characteristics were calculated using the following formulas:

(1) $$Tensile\;strength\;(\mathrm{kg}/{\mathrm{mm}}^{2})=\frac{Force\;at\;breakage\;(\mathrm{kg})}{Film\;thickness\;\left(\mathrm{mm}\right)\times Film\;width\;(\mathrm{mm})}$$

(2) $$Elongation\;(\%)=\frac{Increased\;film\;length}{Inital\;film\;length}\times 100$$

##### Moisture Content

The moisture content was evaluated using an HR 73 Mettler Toledo halogen humidity analyzer, produced by Mettler-Toledo GmbH (Greifensee, Switzerland), using the thermogravimetric technique [91]. It was calculated as the drying loss (%). The measurements were performed in triplicate.

##### Swelling Behavior 

The swelling ability of hydrogel was evaluated by varying pH conditions, from an acidic to a basic value. A total of 3 different mediums were prepared and used in the analysis: HCl/H_2_O solution (pH 3), purified water (pH 7), and NaOH/H_2_O solution (pH 9). In each solution, 0.1 g of hydrogel was placed and kept at room temperature (22 °C). At every hour, the samples were withdrawn and weighed. The swelling behavior was determined at 6 h. The percentages of water absorption were calculated according to Equation (3):

(3) $$Swelling\;ratio\;(\%)=\frac{Wt}{Wi}\times 100$$

where *W_t_* is the sample weight at time *t* after the incubation, and W_i_ is the initial weight [92,93,94]. The experiments were performed in triplicates.

### 4.4. Preliminary Adsorption Studies

Initially, dye adsorption test studies were conducted to preliminarily assess the capacity of adsorption of hydrogel-based material as an adsorbent. As a model for adsorption assays, the following dyes were used: gentian violet, methyl orange, and eosin (purchased from Merck, Germany). For adsorption experiments, 0.5 g of dry hydrogel was added to 10 mL of each dye solution, of concentration 20 mg/L, at room temperature, at pH 7. The evaluation of the visual adsorption was determined after 24 h of contact time.

### 4.5. Batch Adsorption Study

A Carry Varian X100 spectrophotometer was used for the batch adsorption study. The stock solution of DCF sodium salt of 1 mg per 1 mL was prepared with distilled water and stored refrigerated. For adsorption experiments, certain amounts of CPX were weighed out and left for 24 h in 10 mL of water to swell. After that, 0.33 mL of DCF stock solution was added to each sample. At the desired time interval, the solutions containing DCF were scanned in the 200–400 nm UV range using a 1 cm quartz cuvette. The residual concentration of DCF from the aqueous solution was quantified by following the decrease of the adsorption maximum of DCF at λ = 276 nm (ε = 9580 M^−1^ × cm^−1^). 

The DCF removal efficiency and CPX adsorption capacity were estimated using the following equations:

(4) $$Ad\left(\%\right)=\frac{{(C}_{0}-C_{t})\times 100}{C_{0}}$$

(5) $$q_{t}=\frac{\left(C_{0}-C_{t}\right)\times V}{W}$$

(6) $$q_{e}=\frac{\left(C_{0}-C_{e}\right)\times V}{W}$$

where *Ad* (%) is the DCF removal efficiency of CPX; $q_{e}$ and $q_{t}$ are the adsorption capacity expressed in (mg × g^−1^) at equilibrium and at time *t* (min), respectively; $C_{0}$, $C_{t},$ and $C_{e}$ are the initial DCF concentration, the concentration of DCF that is still in the solution at a time *t*, and the equilibrium DCF concentration (mg × L^−1^), respectively; *V* is the volume of the aqueous solution (L); and *W* is the mass of the CPX adsorbent (g).

The curves obtained by plotting the $q_{t}$ function of *t* were fitted by nonlinear regression using an equation that describes a kinetic model specific for liquid–solid phase adsorption, a process of pseudo first order:

(7) $$q_{t}=q_{e}(1-e^{{-k}_{1}t})$$

The equation allowed the obtaining of $k_{1},$ which is the rate constant of the pseudo first-order sorption (min^−1^) and also the $q_{e}$ adsorption capacity at equilibrium (mg × g^−1^).

Furthermore, the Langmuir and Freundlich adsorption models were both employed to correlate the obtained isotherm information.

The linearized Langmuir equation was expressed as:

(8) $$\frac{C_{e}}{q_{e}}=\frac{1}{q_{max}}C_{e}+\frac{1}{bq_{max}}$$

where $q_{max}$ was the maximum monolayer adsorption capacity (mg × g^−1^), and *b* defines the Langmuir adsorption constant (L × mg^−1^).

Using the parameters obtained with Equation (8), the equilibrium parameter (*R*_L_) by means of Equation (9) was obtained [95]:

(9) $$R_{L}=\frac{1}{1+bC_{0}}$$

The Freundlich equation was also used to investigate the adsorption process on CPX at the equilibrium condition. The theoretical Freundlich isotherm was used in the linearized form as:

(10) $$lnq_{e}={lnK}_{F}+\frac{1}{n}{lnC}_{e}$$

where $K_{F}$ and *n* are the Freundlich parameters that are obtained from the graphical representation of $lnq_{e}$ versus ${lnC}_{e}$.

## Figures and Tables

**Table 1:** Pseudo first-order kinetic parameters of DCF adsorption on different masses of CPX hydrogel.

Pseudo First-Order Parameters	[CPX]				
	10 mg	25 mg	50 mg	100 mg	200 mg
k_1_/(min^−1^)	0.018	0.012	0.013	0.004	0.006
q_e_ /(mg × g^−1^)	208.67	202.44	189.93	187.06	175.01
R^2^	0.99	0.99	0.98	0.98	0.99

**Table 2:** Langmuir and Freundlich isotherm parameters.

Langmuir Isotherm Parameters				Freundlich Isotherm Parameters		
q_max_	b	R_L_	R^2^	K_F_	n	R^2^
172.41	0.0014	0.80	0.99	292.33	16.28	0.93

## Data Availability

Not applicable.

## Supplementary Materials

The following supporting information can be downloaded at: https://www.mdpi.com/article/10.3390/gels9060454/s1, Figure S1: The plot of the non-linear form of the pseudo second-order model for different amounts of CPX hydrogel and 0.33 mg DCF; Table S1: Pseudo second-order kinetic parameters of DCF adsorption on different amounts of CPX hydrogel; Figure S2: Langmuir isotherm representation of DCF adsorption in presence of different CPX amounts; Figure S3: Freundlich isotherm representation of DCF adsorption in presence of different CPX amounts.

## References

[1] Du Plessis A. (2022). Persistent degradation: Global water quality challenges and required actions. One Earth.

[2] Estêvão M.D. (2023). Aquatic Pollutants: Risks, Consequences, Possible Solutions and Novel Testing Approaches. Fishes.

[3] Dulsat-Masvidal M., Ciudad C., Infante O., Mateo R., Lacorte S. (2023). Water pollution threats in important bird and biodiversity areas from Spain. J. Hazard. Mater..

[4] Bhuyan A., Ahmaruzzaman M. (2023). Recent advances in new generation nanocomposite materials for adsorption of pharmaceuticals from aqueous environment. Environ. Sci. Pollut. Res..

[5] Kumar M., Sridharan S., Sawarkar A.D., Shakeel A., Anerao P., Mannina G., Sharma P., Pandey A. (2023). Current research trends on emerging contaminants pharmaceutical and personal care products (PPCPs): A comprehensive review. Sci. Total Environ..

[6] Alessandretti I., Rigueto C.V.T., Nazari M.T., Rosseto M., Dettmer A. (2021). Removal of diclofenac from wastewater: A comprehensive review of detection, characteristics and tertiary treatment techniques. J. Environ. Chem. Eng..

[7] Matamoros V., Rodríguez Y., Albaig’es J. (2016). A comparative assessment of intensive and extensive wastewater treatment technologies for removing emerging contaminants in small communities. Water Res..

[8] Sousa J.C.G., Ribeiro A.R., Barbosa M.O., Pereira M.F.R., Silva A.M.T. (2018). A review on environmental monitoring of water organic pollutants identified by EU guidelines. J. Hazard. Mater..

[9] Loganathan P., Vigneswaran S., Kandasamy J., Cuprys A.K., Maletskyi Z., Ratnaweera H. (2023). Treatment Trends and Combined Methods in Removing Pharmaceuticals and Personal Care Products from Wastewater—A Review. Membranes.

[10] Rahman T.U., Roy H., Islam M.R., Tahmid M., Fariha A., Mazumder A., Tasnim N., Pervez M.N., Cai Y., Naddeo V. (2023). The Advancement in Membrane Bioreactor (MBR) Technology toward Sustainable Industrial Wastewater Management. Membranes.

[11] Rikabi A.A.K.K., Chelu B., Mariana, Harabor I., Albu P.C., Segarceanu M., Nechifor G. (2016). Iono-molecular Separation with Composite Membranes I. Preparation and characterization of membranes with polysulfone matrix. Rev. Chimie.

[12] Diaconu I., Gardea R., Cristea C., Nechifor G., Ruse E., Eftimie Totu E. (2010). Removal and recovery of some phenolic pollutants using liquid membranes. Rom. Biotechnol. Lett..

[13] Nechifor A.C., Goran A., Grosu V.-A., Pîrtac A., Albu P.C., Oprea O., Grosu A.R., Pascu D., Pancescu F.M., Nechifor G. (2021). Reactional Processes on Osmium–Polymeric Membranes for 5–Nitrobenzimidazole Reduction. Membranes.

[14] Diaconu I., Aboul-Enein H.Y., Bunaciu A.A., Ruse E., Mirea C., Nechifor G. (2015). Selective separation of acetaminophene from pharmaceutical formulations through membrane techniques. Rev. Roum. Chim..

[15] Serban E.A., Diaconu I., Ruse E., Ghe B., Nechifor G., Lazar M.N. (2018). Bulk Liquid Membranes for Separation and Recovery of Pharmaceutical Products. Rev. Chim..

[16] Renault F., Sancey B., Badot P.M., Crini G. (2009). Chitosan for coagulation/flocculation processes—An eco-friendly approach. Eur. Polym. J..

[17] Bhatt P., Joshi S., Urper Bayram G.M., Khati P., Simsek H. (2023). Developments and application of chitosan-based adsorbents for wastewater treatments. Environ. Res..

[18] Bezerra de Araujo C.M., Ghislandi M.G., Gonçalves Rios A., Bezerra da Costa G.R., do Nascimento B.F., Ferreira A.F.P., da Motta Sobrinho M.A., Rodrigues A.E. (2022). Wastewater treatment using recyclable agar-graphene oxide biocomposite hydrogel in batch and fixed-bed adsorption column: Bench experiments and modeling for the selective removal of organics. Colloids Surf. A Physicochem. Eng. Asp..

[19] Resende J.F., Paulino I.M.R., Bergamasco R., Vieira M.F., Vieira A.M.S. (2023). Hydrogels produced from natural polymers: A review on its use and employment in water treatment. Braz. J. Chem. Eng..

[20] Chelu M., Musuc A.M. (2023). Polymer Gels: Classification and Recent Developments in Biomedical Applications. Gels.

[21] Chelu M., Popa M., Ozon E.A., Pandele Cusu J., Anastasescu M., Surdu V.A., Calderon Moreno J., Musuc A.M. (2023). High-Content Aloe vera Based Hydrogels: Physicochemical and Pharmaceutical Properties. Polymers.

[22] Thakur S., Sharma B., Verma A., Chaudhary J., Tamulevicius S., Thakur V.K. (2018). Recent progress in sodium alginate based sustainable hydrogels for environmental applications. J. Clean. Prod..

[23] Khan S.A., Shah L.A., Shah M., Jamil I. (2022). Engineering of 3D polymer network hydrogels for biomedical applications: A review. Polym. Bull..

[24] Bezerra de Araujo C.M., Wernke G., Ghislandi M.G., Diório A., Vieira M.F., Bergamasco R., da Motta Sobrinho M.A., Rodrigues A.E. (2023). Continuous removal of pharmaceutical drug chloroquine and Safranin-O dye from water using agar-graphene oxide hydrogel: Selective adsorption in batch and fixed-bed experiments. Environ. Res..

[25] Machado T.S., Crestani L., Marchezi G., Melara F., Rafael de Mello J., Dotto G.L., Piccin J.S. (2022). Synthesis of glutaraldehyde-modified silica/chitosan composites for the removal of water-soluble diclofenac sodium. Carbohydr. Polym..

[26] Mottaghi H., Mohammadi Z., Abbasi M., Tahouni N., Panjeshahi M.H. (2021). Experimental investigation of crude oil removal from water using polymer adsorbent. J. Water Process Eng..

[27] Gkika D.A., Mitropoulos A.C., Kokkinos P., Lambropoulou D.A., Kalavrouziotis I.K., Bikiaris D.N., Kyzas G.Z. (2023). Modified chitosan adsorbents in pharmaceutical simulated wastewaters: A review of the last updates. Carbohydr. Polym. Technol. Appl..

[28] Petri D.F.S. (2015). Xanthan gum: A versatile biopolymer for biomedical and technological applications. Appl. Polym. Sci..

[29] Deng S., Wang R., Xu H., Jiang X., Yin J. (2012). Hybrid hydrogels of hyperbranched poly(ether amine)s (hPEAs) for selective adsorption of guest molecules and separation of dyes. J. Mater. Chem..

[30] Zare E.N., Fallah Z., Le V.T., Doan V.-D., Mudhoo A., Joo S.-W., Vasseghian Y., Tajbakhsh M., Moradi O., Sillanpaa M. (2022). Remediation of pharmaceuticals from contaminated water by molecularly imprinted polymers: A review. Environ. Chem. Lett..

[31] Fortunato A., Mba M. (2022). A Peptide-Based Hydrogel for Adsorption of Dyes and Pharmaceuticals in Water Remediation. Gels.

[32] Das B.K., Samanta R., Ahmed S., Pramanik B. (2023). Disulphide Cross-Linked Ultrashort Peptide Hydrogelator for Water Remediation. Chem. Eur. J..

[33] Grohs L., Cheng L., Cönen S., Haddad B.G., Bülow A., Toklucu I., Ernst L., Körner J., Schmalzing G., Lampert A. (2023). Diclofenac and other non-steroidal anti-inflammatory drugs (NSAIDs) are competitive antagonists of the human P2X3 receptor. Front. Pharmacol..

[34] Bonnefille B., Gomez E., Courant F., Escande A., Fenet H. (2018). Diclofenac in the marine environment: A review of its occurrence and effects. Mar. Pollut. Bull..

[35] de Carvalho Filho J.A.A., da Cruz H.M., Fernandes B.S., Motteran F., de Paiva A.L.R., da Silva Pereira Cabral J.J. (2022). Efficiency of the bank filtration technique for diclofenac removal: A review. Environ. Pollut..

[36] Fahimi A., Zanoletti A., Federici S., Assi A., Bilo F., Depero L.E., Bontempi E. (2020). New eco-materials derived from waste for emerging pollutants adsorption: The case of diclofenac. Materials.

[37] Godiya C.B., Kumar S., Xiao Y. (2020). Amine functionalized egg albumin hydrogel with enhanced adsorption potential for diclofenac sodium in water. J. Hazard. Mater..

[38] Khan S.A., Siddiqui M.F., Khan T.A. (2020). Synthesis of Poly(methacrylic acid)/Montmorillonite Hydrogel Nanocomposite for Efficient Adsorption of Amoxicillin and Diclofenac from Aqueous Environment: Kinetic, Isotherm, Reusability, and Thermodynamic Investigations. ACS Omega.

[39] Feng Z., Simeone A., Odelius K., Hakkarainen M. (2017). Biobased Nanographene Oxide Creates Stronger Chitosan Hydrogels with Improved Adsorption Capacity for Trace Pharmaceuticals. ACS Sustain. Chem. Eng..

[40] Umbreen N., Sohni S., Ahmad I., Khattak N.U., Gul K. (2018). Self-assembled three-dimensional reduced graphene oxide-based hydrogel for highly efficient and facile removal of pharmaceutical compounds from aqueous solution. J. Colloid Interface Sci..

[41] Mahmoodi H., Fattahi M., Motevassel M. (2021). Graphene oxide–chitosan hydrogel for adsorptive removal of diclofenac from aqueous solution: Preparation, characterization, kinetic and thermodynamic modelling. RSC Adv..

[42] Zaka A., Ibrahim T.H., Khamis M. (2021). Removal of selected non-steroidal anti-inflammatory drugs from wastewater using reduced graphene oxide magnetite. Desalination Water Treat..

[43] Argin-Soysal S., Kofinas P., Lo Y.M. (2009). Effect of complexation conditions on xanthan–chitosan polyelectrolyte complex gels. Food Hydrocoll..

[44] Corrias F., Dolz M., Herraez M., Diez-Sales O. (2008). Rheological properties of progesterone microemulsions: Influence of xanthan and chitosan biopolymer concentration. J. Appl. Polym. Sci..

[45] Phaechamud T., Ritthidej G.C. (2008). Formulation variables influencing drug release from layered matrix system comprising chitosan and xanthan gum. AAPS PharmSciTech.

[46] Popa N., Novac O., Profire L., Lupusoru C.E., Popa M.I. (2010). Hydrogels based on chitosan–xanthan for controlled release of theophylline. J. Mater. Sci. Mater. Med..

[47] Bellini M.Z., Pires A.L.R., Vasconcelos M.O., Moraes A.M. (2012). Comparison of the properties of compacted and porous lamellar chitosan–xanthan membranes as dressings and scaffolds for the treatment of skin lesions. J. Appl. Polym. Sci..

[48] Luo Y., Wang Q. (2014). Recent development of chitosan-based polyelectrolyte complexes with natural polysaccharides for a drug delivery. Int. J. Biol. Macromol..

[49] Chelu M., Moreno J.C., Atkinson I., Cusu J.P., Rusu A., Bratan V., Aricov L., Anastasescu M., Seciu-Grama A.-M., Musuc A.M. (2022). Green synthesis of bioinspired chitosan-ZnO-based polysaccharide gums hydrogels with propolis extract as novel functional natural biomaterials. Int. J. Biol. Macromol..

[50] Zajac A., Hanuza J., Wandas M., Dyminska L. (2015). Determination of N-acetylation degree in chitosan using Raman spectroscopy. Spectrochim. Acta Part A Mol. Biomol. Spectrosc..

[51] Malik N.S., Ahmad M., Minhas M.U., Tulain R., Barkat K., Khalid I., Khalid Q. (2020). Chitosan/Xanthan Gum Based Hydrogels as Potential Carrier for an Antiviral Drug: Fabrication, Characterization, and Safety Evaluation. Front Chem..

[52] Dey S.C., Al-Amin M., Rashid T.U., Sultan M.Z., Ashaduzzaman M., Sarker M., Shamsuddin S.M. (2016). Reparation, characterization and performance evaluation of chitosan as an adsorbent for remazol red. Int. J. Latest Res. Eng. Technol..

[53] De Morais Lima M., Carneiro L.C., Bianchini D., Dias R.G.A., da Rosa Zavareze E., Prentice C., da Silveira Moreira A. (2017). Structural, Thermal, Physical, Mechanical and Barrier Properties of Chitosan Films with the Addition of Xanthan Gum. J. Food Sci..

[54] Horn M.M., Martins V.C.A., de Guzzi Plepis A.M. (2015). Influence of collagen addition on the thermal and morphological properties of chitosan/xanthan hydrogels. Int. J. Biol. Macromol..

[55] Nirmala R., Il B.W., Navamathavan R., El-Newehy M.H., Kim H.Y. (2011). Preparation and characterizations of anisotropic chitosan nanofibers via electrospinning. Macromol. Res..

[56] Hussien M.A., Ebtessam A.E., El G.S.A. (2019). Investigation of the effect of formulation additives on telmisartan dissolution rate: Development of oral disintegrating tablets. Eur. J. Biomed. Pharm. Sci..

[57] Essa E., Elmarakby A., Donia A., El Maghraby G.M. (2017). Controlled precipitation for enhanced dissolution rate of flurbiprofen: Development of rapidly disintegrating tablets. Drug Dev. Ind. Pharm..

[58] Kuzmin V.V., Novikov V.S., Ustynyuk L.Y., Prokhorov K.A., Sagitova E.A., Nikolaeva G.Y. (2020). Raman spectra of polyethylene glycols: Comparative experimental and DFT study. J. Mol. Struct..

[59] Matsuura H., Fukuhara K. (1985). Conformational analysis of poly(oxyethylene) chain in aqueous solution as a hydrophilic moiety of nonionic surfactants. J. Mol. Struct..

[60] Matsuura H., Fukuhara K. (1986). Vibrational spectroscopic studies of conformation of poly(oxyethylene). II. Conformation–spectrum correlations. J. Polym. Sci. Part B Polym. Phys..

[61] Takahashi Y., Tadokoro H. (1973). Structural studies of polyethers, (-(CH2)m-O-)n. X. crystal structure of poly(ethylene oxide). Macromolecules.

[62] Kumar S., Dutta P.K., Koh J. (2011). A physiocochemical and biological study of novel chitosan-chloroquinoline derivative for biomedical applications. Int. J. Biol. Macromol..

[63] Podgorbunskikh E., Kuskov T., Rychkov D., Lomovskii O., Bychkov A. (2022). Mechanical Amorphization of Chitosan with Different Molecular Weights. Polymers.

[64] Liu Z., Fu X., Jiang L., Wu B., Wang J., Lei J. (2016). Solvent-free synthesis and properties of novel solid–solid phase change materials with biodegradable castor oil for thermal energy storage. Sol. Energy Mater. Sol. Cells.

[65] Fița A.C., Secăreanu A.A., Musuc A.M., Ozon E.A., Sarbu I., Atkinson I., Rusu A., Mati E., Anuta V., Pop A.L. (2022). The Influence of the Polymer Type on the Quality of Newly Developed Oral Immediate-Release Tablets Containing Amiodarone Solid Dispersions Obtained by Hot-Melt Extrusion. Molecules.

[66] Kang Y., Li P., Zeng X., Chen X., Xie Y., Zeng Y., Zhang Y., Xie T. (2019). Biosynthesis, structure and antioxidant activities of xanthan gum from Xanthomonas campestris with additional furfural. Carbohydr. Polym..

[67] Cheung M.K., Wan K.P., Yu P.H. (2002). Miscibility and morphology of chiral semicrystalline poly-(R)-(3-hydroxybutyrate)/chitosan and poly-(R)-(3-hydroxybutyrate-co-3-hydroxyvalerate)/chitosan blends studied with DSC,1HT1 andT1? CRAMPS. J. Appl. Polym. Sci..

[68] Kittur F., Prashanth K.H., Sankar K.U., Tharanathan R. (2002). Characterization of chitin, chitosan and their carboxymethyl derivatives by differential scanning calorimetry. Carbohydr. Polym..

[69] Cardenas G., Miranda S.P. (2004). FTIR and TGA studies of chitosan composite films. J. Chil. Chem. Soc..

[70] Kacurakova M., Belton P.S., Hirsch J., Ebringerova A. (1998). Hydration Properties of Xylan-Type Structures: An Study of Xylo-oligosaccharides FTIR. J. Sci. Food Agric..

[71] Phillips G.O., Takigami S., Takigami M. (1996). Hydration characteristics of the gum exudate from Acacia Senegal. Food Hydrocoll..

[72] Appelquist I.A.M., Cooke D., Gidley M.J., Lane S.J. (1993). Effect of Heat Treatment on the Pectins of Tomatoes during Tomato Paste Manufacturing. Carbohydr. Polym..

[73] Gidley M.J., Robinson G. (1990). 18—Techniques for Studying Interactions Between Polysaccharides. Methods Plant Biochem..

[74] Sreenivasan K. (1996). Thermal stability studies of some chitosan metal ion complexes using differential scanning calorimetry. Polym. Degrad. Stab..

[75] Deng L., Qi H., Yao C., Feng M., Dong A. (2007). Investigation on the properties of methoxy poly (ethylene glycol)/chitosan graft co-polymers. J. Biomater. Sci. Polym. Ed..

[76] Nunes M.M., Menezes P.F., Alves R.M., Gonçalves R.J., Junges A., Formentin M.W., Mendonça D.F., Ligabue R.A., Bueno M.F., Severino P. (2021). Chitosan and chitosan/PEG nanoparticles loaded with in-dole-3-carbinol: Characterization, computational study and potential effect on human bladder cancer cells. Mater. Sci. Eng. C.

[77] Jayaramudu T., Raghavendra G.M., Varaprasad K., Reddy G.V.S., Reddy A.B., Sudhakar K., Sadiku E.R. (2016). Preparation and characterization of poly(ethylene glycol) stabilized nano silver particles by a mechanochemical assisted ball mill process. J. Appl. Polym. Sci..

[78] Yang J.H., Han Y.S., Park M., Park T., Hwang S.J., Choy J.H. (2007). New inorganic-based drug delivery system of indole-3-acetic acid-layered metal hydroxide nanohybrids with controlled release rate. Chem. Mater..

[79] Chitra G., Franklin D.S., Guhanathan S. (2017). Indole-3-acetic acid based tunable hydrogels for antibacterial, antifungal and antioxidant applications. J. Macromol. Sci. Part A Pure Appl. Chem..

[80] Yongmei G., Chengqun Y., Zhenzhong Z., Xinhao W., Abid N., Rui Z., Weifeng Z. (2023). Chitosan/xanthan gum-based (Hydroxypropyl methylcellulose-co-2-Acrylamido-2-methylpropane sulfonic acid) interpenetrating hydrogels for con-trolled release of amorphous solid dispersion of bioactive constituents of Pueraria lobatae. Int. J. Biol. Macromol..

[81] Kulkarni N., Wakte P., Naik J. (2015). Development of floating chitosan-xanthan beads for oral controlled release of glipizide. Int. J. Pharm. Investig..

[82] Nafisa G., Shahzad M.K., Osama M.B., Atif I., Attaullah S., Sehrish J., Saba U.K., Afrasyab K., Rafi U.K., Muhammad T.Z.B. (2020). Inflammation targeted chitosan-based hydrogel for controlled release of diclofenac sodium. Int. J. Biol. Macromol..

[83] Lessa E.F., Nunes M.L., Fajardo A.R. (2018). Chitosan/waste coffee-grounds composite: An efficient and eco-friendly adsorbent for removal of pharmaceutical contaminants from water. Carbohydr. Polym..

[84] Martinez-Ruvalcaba A., Chornet E., Rodrigue D. (2007). Viscoelastic properties of dispersed chitosan/xanthan hydrogels. Carbohydr. Polym..

[85] Hu D., Huang H., Jiang R., Wang N., Xu H., Wang Y.G., Ouyang X.K. (2019). Adsorption of diclofenac sodium on bilayer amino-functionalized cellulose nanocrystals/chitosan composite. J. Hazard. Mater..

[86] Liang X.X., Omer A.M., Hu Z.-H., Wang Y.G., Di Y., Ouyang X.-K. (2019). Efficient adsorption of diclofenac sodium from aqueous solutions using magnetic amine-functionalized chitosan. Chemosphere.

[87] Li S., Cui J., Wu X., Zhang X., Hu Q., Hou X. (2019). Rapid in situ microwave synthesis of Fe3O4@MIL-100(Fe) for aqueous diclofenac sodium removal through integrated adsorption and photodegradation. J. Hazard. Mater..

[88] Zhuang S., Cheng R., Wang J. (2019). Adsorption of diclofenac from aqueous solution using UiO-66-type metal-organic frameworks. Chem. Eng. J..

[89] Xiong T., Yuan X., Wang H., Wu Z., Jiang L., Leng L., Xi K., Cao X., Zeng G. (2019). Highly efficient removal of diclofenac sodium from medical wastewater by Mg/Al layered double hydroxide-poly(m-phenylenediamine) composite. Chem. Eng. J..

[90] Zhao R., Zheng H., Zhong Z., Zhao C., Sun Y., Huang Y., Zheng X. (2021). Efficient removal of diclofenac from surface water by the functionalized multilayer magnetic adsorbent: Kinetics and mechanism. Sci. Total Environ..

[91] Balaci T., Velescu B., Karampelas O., Musuc A.M., Nitulescu G.M., Ozon E.A., Nitulescu G., Gird C.E., Fita C., Lupuliasa D. (2021). Physico-Chemical and Pharmaco-Technical Characterization of Inclusion Complexes Formed by Rutoside with beta-Cyclodextrin and Hydroxypropyl-beta-Cyclodextrin Used to Develop Solid Dosage Forms. Processes.

[92] Nafee N.A., Ismail F.A., Boraie N.A., Mortada L.M. (2003). Mucoadhesive buccal patches of miconazole nitrate: In vitro/in vivo performance and effect of ageing. Int. J. Pharm..

[93] Don T.M., Huang M.L., Chiu A.C. (2008). Preparation of thermo-responsive acrylic hydrogels useful for the application in transdermal drug delivery systems. Mater Chem Phys.

[94] Wang K., Fu Q., Chen X., Gao Y., Dong K. (2012). Preparation and characterization of pH-sensitive hydrogel for drug delivery system. RSC Adv..

[95] Yi Z., Yao J., Zhu M., Chen H., Wang F., Liu X. (2016). Kinetics, equilibrium, and thermodynamics investigation on the adsorption of lead (II) by coal-based activated carbon. SpringerPlus.

