# Supplementary material for: Green Synthesis of Hydrogel-Based Adsorbent Material for the Effective Removal of Diclofenac Sodium from Wastewater
Source: Gels. 2023 Jun 1;9(6):454. doi: 10.3390/gels9060454 (PMC10297473; doi:10.3390/gels9060454)
Supplement: Supplementary file 1 [file gels-09-00454-s001.zip › gels-2400687-supplementary.pdf]

Supplementary Materials file

Green Synthesis of Hydrogel-Based Adsorbent Material for the Effective Removal of Diclofenac Sodium from Wastewater

Mariana Chelu [1,*,†], Monica Popa [1,†], Jose Calderon Moreno [1,*], Anca Ruxandra Leonties [1], Emma Adriana Ozon [2], Jeanina Pandele Cusu [1], Vasile Adrian Surdu [3], Ludmila Aricov [1] and Adina Magdalena Musuc [1,*]

[1] "Ilie Murgulescu" Institute of Physical Chemistry, 202 Spl. Independentei, 060021 Bucharest, Romania; pmonica@icf.ro (M.P.); aleonties@icf.ro (A.R.L.); jeanina@icf.ro (J.P.C); laricov@icf.ro (L.A.)

[2] Department of Pharmaceutical Technology and Biopharmacy, Faculty of Pharmacy, Carol Davila University of Medicine and Pharmacy, 6 Traian Vuia Street, 020945 Bucharest, Romania. emma.budura@umfcd.ro (E.A.O.)

[3] Department of Science and Engineering of Oxide Materials and Nanomaterials, Faculty of Applied Chemistry and Materials Science, University Politehnica of Bucharest, 060042 Bucharest, Romania; adrian.surdu@upb.ro (V.A.S.)

[*] Correspondence: mchelu@icf.ro (M.C.); calderon@icf.ro (J.C.M.); amusuc@icf.ro (A.M.M.).

[†] These authors contributed equally to this work.

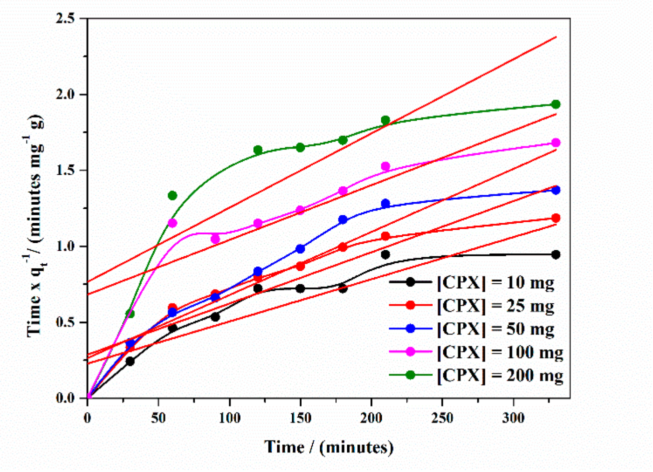

Figure S1. The plot of the non-linear form of the pseudo second-order model for different amounts of CPX hydrogel and 0.33 mg DCF

Table S1. Pseudo second-order kinetic parameters of DCF adsorption on different amounts of CPX hydrogel.

[CPX]/(mg)	Intercept	Slope	Adj. R^2
10	0.23	2.78×10^{-3}	0.78
25	0.29	3.37×10^{-3}	0.81
50	0.26	4.15×10^{-3}	0.86
100	0.77	4.88×10^{-3}	0.50
200	0.68	3.60×10^{-3}	0.53

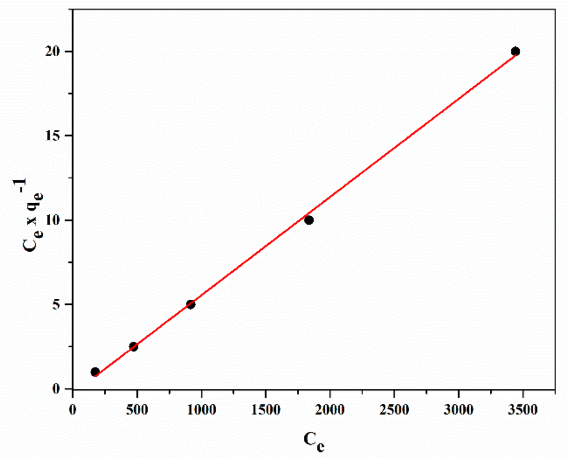

Figure S2. Langmuir isotherm representation of DCF adsorption in presence of different CPX amounts.

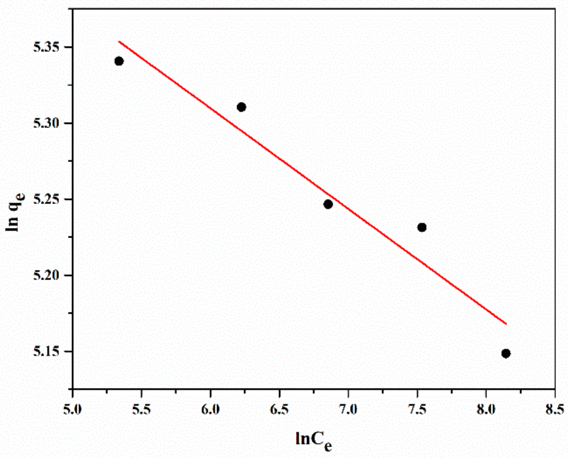

Figure S3. Freundlich isotherm representation of DCF adsorption in presence of different CPX amounts.
